# Techniques of using circulating tumor DNA as a liquid biopsy component in cancer management

**DOI:** 10.1016/j.csbj.2018.10.002

**Published:** 2018-10-09

**Authors:** Maha Elazezy, Simon A. Joosse

**Affiliations:** University Medical Center Hamburg-Eppendorf, Martinistr. 52, 20246 Hamburg, Germany

**Keywords:** Liquid biopsy, Circulating tumor DNA (ctDNA), Cell-free DNA (cfDNA)

## Abstract

Precision medicine in the clinical management of cancer may be achieved through the diagnostic platform called “liquid biopsy”. This method utilizes the detection of biomarkers in blood for prognostic and predictive purposes. One of the latest blood born markers under investigation in the field of liquid biopsy in cancer patients is circulating tumor DNA (ctDNA). ctDNA is released by tumor cells through different mechanisms and can therefore provide information about the genomic make-up of the tumor currently present in the patient. Through longitudinal ctDNA-based liquid biopsies, tumor dynamics may be monitored to predict and assess drug response and/or resistance. However, because ctDNA is highly fragmented and because its concentration can be extremely low in a high background of normal circulating DNA, screening for clinical relevant mutations is challenging. Although significant progress has been made in advancing the detection and analysis of ctDNA in the last few years, the current challenges include standardization and increasing current techniques to single molecule sensitivity in combination with perfect specificity. This review focuses on the potential role of ctDNA in the clinical management of cancer patients, the current technologies that are being employed, and the hurdles that still need to be taken to achieve ctDNA-based liquid biopsy towards precision medicine.

## 1 Introduction

Cancer is the consequence of deregulation of tumor suppressors and proto-oncogenes caused by the accumulation of mutations in the genome of a normal cell [1,2]. Proto-oncogenes promote cell division and proliferation, whereas tumor suppressors can induce apoptosis and are negative regulators of cell proliferation [3]. The identification of the genetic and/or epigenetic modifications leading to pathogenesis can be exploited for anticancer therapy management, prediction, and prognosis [4]. Cancer-related mutations include chromosomal aberrations such as copy numbers alterations (CNAs), inversions, translocations, insertions, and deletions, as well as single nucleotide point mutations [3]. Epigenetics refers to the covalent modification of DNA resulting in changes to the function and/or regulation of the affected genes, without altering the primary sequences (a change in phenotype without a change in genotype). Epigenetic factors such as DNA methylation and histone modification, play a key role in gene activity, cell differentiation, tumorigenesis, X-chromosome inactivation, genomic imprinting, and other cellular regulatory processes [5].

**Fig. 1:**
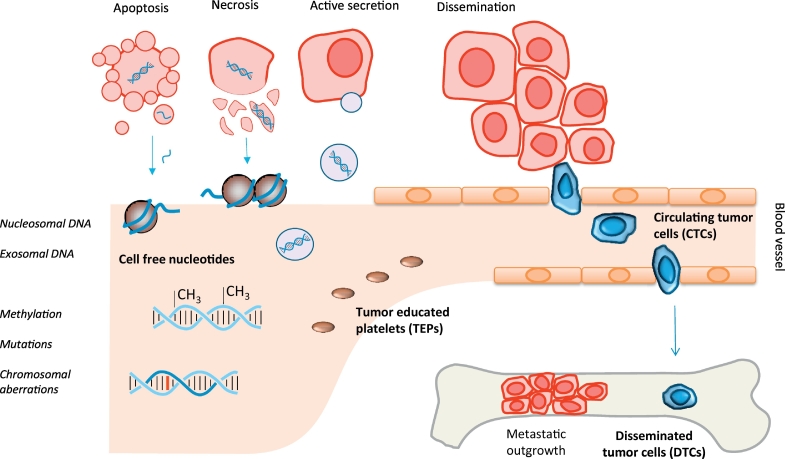
Liquid biopsy markers. Biomarkers that are currently used as liquid biopsy include cell free nucleotides, circulating tumor cells (CTCs), tumor educated platelets (TEPs), and disseminated tumor cells (DTCs). Cell free nucleotides are released into the blood circulating by apoptotic or necrotic cells, or by active secretion of exosomes containing a cell's genetic material. Cell free DNA (cfDNA) is highly fragmented but is still wrapped around nucleosomes providing its typical length of 166 or 320 bp. cfDNA may be used to study a tumor's methylation patterns, chromosomal aberrations, or other mutations.

Metastatic spread is the main cause of cancer-related death and is the result of colonization of tumor cells from the primary tumor into distant organs, which may finally be followed by organ failure. The route of dissemination takes place mainly through the blood circulation, in which only very few circulating tumor cells (CTCs) are able to survive [6]. Extravasation of the tumor cells is usually expected to occur in distant organs such as the brain, bone marrow, lungs, or liver in which the disseminated tumor cell (DTCs) can stay dormant for many years (Fig. 1) [7]. The observation of DTCs in bone marrow has been shown to be highly correlated with recurrence of disease [8].

In order to molecularly characterize the tumor and identify potential therapeutic targets, material directly taken from the tumor has to be investigated. The standard procedure to genotype a tumor is by obtaining a small piece of tissue using a tissue biopsy, which is a rather invasive procedure. Furthermore, neoadjuvant treatment may shrink the tumor to undetectable size, leaving no tissue for further investigation. Therefore, the procedure to obtain a tissue biopsy is severely hampered by spatial and temporal limitations; in addition, a single biopsy sample may not represent the full tumor load's heterogeneity [9,10]. As an alternative to characterize the tumor, blood can be used to obtain biomolecules or other markers originating from the tumor. One of these markers is circulating tumor cells (CTCs) that originate from the currently present tumor and thereby can function as a so-called “liquid biopsy” (Fig. 1) [11].

The identification of CTCs has been shown to have prognostic and predictive value in different entities of early-stage cancer [12]. However, highly sensitive techniques are required to identify the small number of cells in the extremely high background of normal cells. The different methods available for obtaining CTCs are either based on specific cellular makers expressed on the cell surface [13] or on the physical properties of the cells. Antigens expressed by the tumor cells enable positive enrichment whereas negative enrichment can be achieved by depletion of white blood cells [6]. Because the half-life time of CTCs is <2.5 hours [14] and the metastases are also able to shed tumor cells into the circulation, more CTCs can be expected in the advanced stages of the disease [15]. Other blood-borne biomarkers currently used as liquid biopsy include platelets, cell-free nucleotides, and extracellular vesicles such as exosomes (Fig. 1) [11]. Platelets may be altered through confrontation with tumor cells via transfer of tumor-associated biomolecules [16]. These so called tumor-educated platelets (TEPs) contain a variety of RNA transcripts and proteins that may influence the process of metastasis development by enhancing or blocking tumor cells, immune cells, and stromal cells, either by direct cell-to-cell contact or by releasing extracellular queues [17,18]. Exosomes are an effective way for cells to secrete mRNA and miRNA into the circulation that may lead to disease progression [19]. For example, exosome-mediated transfer of cancer-secreted miR-105 promotes metastasis in breast cancer [20]. Therefore, identification of such cell-free miRNAs can be used to serve as a biomarker for the early stage of metastasis [21]. Besides RNA, cell-free nucleotides also include cell-free DNA (cfDNA). As a consequence, liquid biopsy may also include the screening for fetal aneuploidy where the cfDNA originates either from the fetus or from apoptotic placental cells, circulating in a pregnant woman's plasma, is investigated [22]. This review will focus on the use of cfDNA originating from the tumor, i.e., circulating tumor DNA (ctDNA), for the clinical management of cancer patients and provide a comprehensive overview of the different techniques being applied to obtain and characterize ctDNA.

## 2 Circulating tumor DNA (ctDNA) properties

Two processes are involved in the release of ctDNA into the blood circulating [23]. The first is a passive release of DNA through cell death either by apoptosis or necrosis (Fig. 1). As a consequence of enzymatic cleavage of DNA during apoptosis, the resulting DNA fragments are still wrapped around single nucleosomes and the length plus linker is around 166 bp [24,25]. Larger fragments starting from 320 bp, the length of DNA wrapped around two nucleosomes, up to >1000 bp are released from phagocytosis of necrotic cells [23]. The second mechanism of ctDNA release is by active secretion [23]. Secretion of ctDNA takes place by the release of extracellular vesicles, such as exosomes and prostasomes, containing pieces of DNA around 150-250 bp [26]. Plasma DNA that originates specifically from tumors (ctDNA) typically represents 0.01–90% of the total cell-free DNA (cfDNA) found in blood [27,28]. It is hypothesized that ctDNA is secreted by tumor cells as a signaling molecule to drive tumor metastasis [29,30]. For example, two independent studies demonstrated that ctDNA may be involved in tumorigenesis and metastasis development. By incubating murine NIH-3 T3 cells with plasma from patients with *KRAS* mutated colorectal tumors followed by injection into mice, the development of tumors could subsequently be observed as well as the detection of human *KRAS* mutations in the mice’ plasma [29,30]. Furthermore, it was observed that ctDNA could promote the proliferation of hormone receptor-positive breast cancer cells by activation of the TLR9-NF-κB-cyclin D1 pathway in vitro [31]. Finally, a small part of the ctDNA may originate from CTCs that die in the blood stream [32].

The rate of ctDNA shedding into the circulation depends on the location, size, and vascularity of the tumor, leading to a difference in ctDNA levels among patients [33,34]. The half-life time of ctDNA in the blood circulation ranges from 16 minutes to 2.5 hours [35]. The concentration of the total cfDNA in healthy individuals is on average 30 ng/ml plasma and ranges from 0 to 100 ng/ml, whereas in cancer patients this can be up to 1000 ng/ml [36,37]. In order to extract cfDNA from the blood, different methods have been developed. Magnetic enrichment of cfDNA can be achieved by positively charged magnetic beads that bind the negatively charged phosphate backbone of DNA [[38], [39], [40], [41]], whereas silica column-based enrichment makes use of the binding affinity of DNA molecules [[38], [39], [40],[42], [43], [44]]. Furthermore, cfDNA capturing can be performed by polymer mediated enrichment (PME) [39] or by a phenol-chloroform based extraction procedure in which DNA is not soluble [42]. Several studies have compared these extraction methods using DNA yield, fragment size distribution, and the quality of the obtained DNA in downstream analysis using for instance mutation detection as a read-out [38,39,42,43]. However, these studies have shown large variations in cfDNA yield and/or fragment size between the different extraction methods. For example, conventional extraction methods based on phenol-chloroform have shown higher yields than with DNA extraction kits, but DNA purity and thereby efficiency of downstream analyses was lower as compared to the magnetic-based method [40]. Some studies have favored the silica-based membrane method due to the high recovery of 82%–92% cfDNA from serum [45]. However, the silica-based membrane system has the disadvantages of a low yield and partial loss of DNA fragments smaller than 150 bp [46,47]. In contrast, a magnetic bead-based method seems to be more efficient in the recovery of short cfDNA fragments as compared to the silica-based membrane and conventional methods [48].

## 3 Clinical applications of ctDNA

The investigation of biomarkers that may help to detect cancer in its early stages before becoming clinically apparent could eventually lead to a decreased mortality [49]. The quantification of cfDNA concentration has been studied to discriminate between healthy individuals and malignant disease [50,51]. It was demonstrated that the levels of cfDNA in NSCLC cancer patients are significantly higher than in healthy individuals [50], in fact, a cutoff level of cfDNA >0.20 mg/ml is able to distinguish between lung cancer patients and control cases with a sensitivity of 69–79% and a specificity of 83–89% [50,51]. Furthermore, many studies have demonstrated that the cfDNA concentration is associated with tumor volume leading to shorter overall survival (OS) of patients with breast [52], ovarian [53], lung [54,55], gastric [56], and colorectal cancer [35,57]. Interestingly, contradictory data have also been reported showing that the concentration of cfDNA did not seem to be associated with overall or progression-free survival [58]. Although, these data indicate that cfDNA levels can be used to monitor tumor progression, using cfDNA for diagnostic purposes is still of limited value.

Quantification of tumor-specific mutations in ctDNA appears to be more relevant for studying tumor progression. High levels of mutated *PIK3CA* in serum DNA of breast cancer patients are associated with short progression-free and overall survival as compared to patients with low or no detectable amounts of mutated ctDNA [59]. The analysis of single nucleotide variants in *KRAS*, *NRAS*, *PIK3CA*, *BRAF*, and *EGFR* using cfDNA has been shown to have >80% concordance when compared to tumor tissue of colorectal [60,61], lung [34,62], and breast [59,61] cancer patients. However, also the time-point at which liquid biopsy is performed in order to track minimal residual disease (MRD) seem to be important, as the ctDNA concentration may lay below the detection limit during certain stages of the treatment. For example, Murillas et al. demonstrated that the detection of ctDNA eight months after surgery is associated with a high risk of relapse in early-stage breast cancer patients, whereas this could not be discerned before the primary surgery based on the detected mutations [63].

ctDNA can also be used to monitor therapy efficiency by detecting mutation-driven resistance [61,64,65]. For example, early detection of *ESR1* mutations*,* which drive endocrine therapy resistance, may help to improve the outcome of patients by switching to other treatment before clinical progression of metastatic breast cancer patients [66]. Likewise, the detection of *KRAS* gene mutations in ctDNA of colorectal cancer patients may indicate resistance to epidermal growth factor receptor inhibitors [61]. Furthermore, decreasing sensitivity to tyrosine kinase inhibitors (TKIs) in patients with gastrointestinal stromal tumors could be demonstrated by tracking primary and secondary hotspot mutations in *KIT* (S821F*)* and *PDGFRA* (D842V) [67]. These data demonstrate the potential of ctDNA to detect and monitor the clonal evolution of cancer through serial genotyping, giving a more complete picture of the distinct genetic subclones that are related to drug resistance [68].

Methylation patterns found on ctDNA can be exploited as biomarkers to detect epigenetic deregulation of genes. Hypermethylation of the promoter of *RASSF1A*, *FHIT*, and *APC* found in plasma DNA was shown to be a useful diagnostic marker for early stage renal cancer with a sensitivity of 56.8% and specificity of 96.7% [69]. The detection of hypermethylation of the *MLH1* gene promoter in ctDNA could be employed as a predictive biomarker for acquired resistance in ovarian cancer and was associated with a poor overall and progression-free survival [70]. Similarly, the identification of methylation of *ESR1* promoter in ctDNA was found to be associated with a lack of response to everolimus/exemestane therapy in metastatic breast cancer patients [71]. Taken together, ctDNA has a high potential for monitoring clinically relevant cancer-related genetic and epigenetic modifications for discovering more detailed information on the tumor characterization [72].

## 4 ctDNA detection technologies

cfDNA is highly fragmented DNA and the total amount of ctDNA might make up as low as 0.01% of the total cfDNA. These extreme low concentrations make the detection challenging, particularly at the early stages of tumor development [27,73,74]. Two strategies have emerged to study the tumor's genomic material by liquid biopsy. First, targeted approaches in which a single or few tumor-specific mutations known from the primary tumor are used for monitoring residual disease in the peripheral blood. Such techniques include Q-PCR, BEAMing, Safe-SeqS, CAPP-Seq, and TAmSeq [57]. The disadvantage of this strategy is that it requires detailed information about the tumor genome. However, targeted monitoring can be extremely sensitive, as mutations can be detected at an allele frequency of down to 0.01% with high specificity and at a fast and cost-effective rate [[75], [76], [77]]. The second strategy to investigate ctDNA involves untargeted screening and aims at a genome-wide analysis for copy number aberrations (CNAs) [78] or point mutations by whole-genome sequencing (WGS) or whole exome sequencing (WES) [79]. Advantages of untargeted strategies include (i) its ability to identify novel changes occurring during tumor treatment and (ii) prior information about the primary tumor's genome is not required. However, a disadvantage is that high concentrations of ctDNA are required for reliable reconstruction of tumor-specific genome-wide changes. Furthermore, untargeted approaches show an overall low sensitivity (5%–10%) [79]. Depending on which strategy is required to investigate the ctDNA or interest, different technologies are currently available (Table 1).

**Table 1:** Technologies for detecting circulating tumor DNA (ctDNA).

Technology	Platform	1-Sensitivity	Specificity	cfDNA input	Number of targets	Type of alteration	Limitations	References
NGS	Deep sequencing (>10,000×)	0.02%	80–90%	2 ng	Panel	Genome-wide copy number changes	Unable to detect rearrangements without assay customization	[[82], [83], [84]]
	TAm-Seq	0.02%	99.9997%	0.9-20 ng	Panel	Known point mutations	Detects only known mutations	[89]
	Safe-SeqS	0.1%	98.9%	3 ng	Panel	Known point mutations and copy number variations	Less comprehensive than WES	[90,91]
	FASTSeqS	>10%	80%	5-10 ng	Panel	Genome-wide copy number changes	Low sensitivity and specificity	[86,87]
	CAPP-Seq	0.004%	>99.99%	32 ng	Panel	Known point mutations, copy number variations, and rearrangements	High cfDNA input; detects only known mutations	[[92], [93], [94]]
	MCTA-Seq	0.25%	89%	7.5 pg	Panel	Known methylation sites		[130]
	Bias-Corrected Targeted NGS	>0.4%	100%		Panel	Known point mutations, copy number variations, and rearrangements		[81]
	Multiplex-PCR NGS	>0.1%	99.6%	2-50 ng	Panel	Known point mutations	Detects only known mutations	[85]
Digital-PCR	ddPCR	0.1%	100%	25 ng	1 to 3	Known point mutations	Detects specific genomic loci; limited in multiplexing	[78,[109], [110], [111]]
	BEAMing	0.01%	100%	1 ng	1 to 20	Known point mutations	Detects only known mutations	[[112], [113], [114], [115]]
Real-Time PCR	AS-PCR	1%	98%	3–50 ng	1	Known point mutations	Low sensitivity; detects known mutations	[[119], [120], [121]]
	AS-NEPB-PCR	0.1%	100%	20 ng	1	Known point mutations	Detects only known point mutations	[76]
	(PNA-LNA) PCR clamp	0.1–1%	79%	30 ng	1	Known point mutations	Low specificity; detects only known point mutations	[[122], [123], [124]]
	(COLD-PCR)	0.1%	94.9%	1–10 ng	1–3	Known point mutations	Detect limited genomic loci; limited in multiplexing	[77]
	MS-PCR	0.62%	100%	20–100 ng	1	Known methylation sites	Detects only specific CpG islands	[71]
Mass-spectrometry technology	SERS	0.1%	100%	5 ng	3 to 10	Known point mutations	Detect limited genomic loci	[125]
	UltraSEEK	0.1%	100%	9 pg-4.2 ng	Up to 40	Known point mutations	Detect limited genomic loci	[126,127]

The performance of the different technologies for detecting ctDNA using different platforms. These technologies differ in sensitivity, specificity, the minimum input of cfDNA, the number of targets that can be analyzed in one reaction, and the type of alterations that can be detected. In addition, the limitations of each technology are indicated. Smallest allele frequencies = 1-sensitivity; TAm-Seq: Tagged-amplicon deep sequencing; Safe-SeqS: Safe-Sequencing System; WES: whole exome sequencing; CAPP-Seq: Cancer Personalized Profiling by deep sequencing; ddPCR: Droplet Digital polymerase chain reaction; BEAMing: Beads, Emulsion, Amplification and Magnetics; AS-PCR: Allele-specific amplification; AS-NEPB-PCR: Allele-Specific, Non-Extendable Primer Blocker PCR; (PNA-LNA) PCR clamp: Peptide Nuclei Acid-Locked Nucleic Acid; COLD-PCR: co-amplification at lower denaturation temperature; MS-PCR: methylation-specific PCR; SERS: surface-enhanced Raman spectroscopy.

An additional strategy might be an alternative to “genotype-independent approaches” a non-invasive screening approach, which based on the fragmentation patterns of an individual's cfDNA that can include an evidence of the epigenetic profile of the origin cells. Such a footprint of nucleosome-bound cfDNA that can be used to determine the contributing cell types in the absence of genotypic differences [80].

### 4.1 Next-generation sequencing (NGS)

NGS has emerged in the past decade as an efficient technique for sequencing DNA and obtaining genetic information. NGS is based on the analysis of several millions of short DNA sequences in parallel followed by either sequence alignment to a reference genome or de novo sequence assembly. Despite its high sensitivity and specificity, NGS shows a random error rate between 0.1% and 1% depending on the applied platform [79] making the detection of ctDNA by rare mutations in the total cfDNA challenging. According to this observation, many protocols have been modified to improve and expand the detection of rare mutations [81] (Table 1).

Deep-sequencing is considered the first approach to detect mutations at an allele-frequency as low as <0.2% by sequencing the target regions with high coverage (>10,000×) [[82], [83], [84]]. As a result, the sensitivity of deep sequencing of finding mutations in cfDNA earlier discovered in tumor tissue can be up to 100%, although the specificity can be as low as 80% [83]. In early stage lung cancer patients (stages IA–IIIA), it was shown that deep sequencing for ctDNA resulted in a low sensitivity of 36.5% in detecting the *EGFR* (L858R) mutation present in the tumor tissue, whereas this increased to 72.7% in metastatic setting (stages IIIB–IV) [84]. The main advantage of deep sequencing is the ability to assess multiple biomarkers simultaneously while its disadvantage is the extreme high read depth that has to be performed in order to detect mutations at low allele frequency and thereby drastically increasing sequencing costs.

Bias-Corrected Targeted NGS is adapted to minimize PCR artifacts by using multifunctional adapters that facilitate read analysis and identify which probe captured the fragment. Bias-Corrected Targeted NGS was applied on cfDNA of NSCLC patients resulting in a detection of >0.4% mutant allele frequency with a specificity of 100% [81]. This technology showed a high specificity in the detection of genomic alterations without producing false positives.

Multiplex-PCR NGS is based on a designed PCR assay panel that facilitates amplification of specific target regions. Validation of the multiplex-PCR NGS platform on the early stage of lung cancer patients showed a highly sensitive detection of >99% of single-nucleotide variants (SNVs) at allele frequencies of >0.1% with a specificity of 99.6% with as less as 20 ng of cfDNA as input material [85].

FAST-SeqS is a simple and efficient method for the detection of aneuploidy by massive parallel sequencing [86,87]. FAST-SeqS can amplify approximately 38,000 amplicons with a single primer pair. During amplification, degenerate bases at the 5′-end of the primer are used as molecular barcodes to uniquely label each DNA template molecule. This ensures that each DNA template molecule is counted only once [88]. A modified version of FAST-SeqS (mFAST-SeqS) was established as a prescreening tool to estimate the ctDNA percentage by using a single primer pair to select and amplify distinct sections of the genome that occur on every chromosome and estimate a genome-wide z-score to evaluate the ctDNA percentage [75]. mFAST-SeqS has for example been used to monitor changing levels of ctDNA in prostate cancer patients before and after treatment, showing a decrease in the genome z-score in patients who responded to therapy [87]. The advantages of this approach include speed (<1 day) and it does not depend on prior knowledge of the genetic composition of tumor samples. Nevertheless, the lowest detection limit of 10% ctDNA is a clear disadvantage [87].

TAm-Seq (Tagged-amplicon deep sequencing) is based on a combination of efficient library preparation and statistically-based analysis algorithms. This technique is adapted to sequence, detect, and quantify tumor mutations across a gene panel including both tumor hotspots, as well as entire coding regions of selected genes [73]. The precision of this methods could be shown by the detection limit of 0.02% with 99.9997% specificity for point mutations in *EGFR* in circulating DNA [89]. The development of a bioinformatic method is a clear advantage that has helped to design more efficient gene panels, improve the detection sensitivity of mutant alleles, and reduce the detection of false positives.

Safe-SeqS was designed to further improve the sensitivity of NGS. Safe-SeqS includes two main steps, the first is to assign a unique identifier (UID) to each DNA template molecule and the second is to amplify each uniquely tagged template to create UID families and sequences [90]. The Safe-SeqS approach has for instance been applied to ctDNA of patients with metastatic colorectal and gastrointestinal stromal tumors (GIST) for tracking therapy response. Here, Safe-SeqS showed a highly sensitive detection of a mutant allele with a concentration of only 0.1% and with a specificity of 98.9% [91,92].

CAncer Personalized Profiling by deep Sequencing (CAPP-Seq) was developed to detect extremely low concentrations of ctDNA by the use of “selectors” consisting of biotinylated DNA oligonucleotides that are complementary to previously defined recurrent mutated areas. Hybridization of the “selectors” on the area-of-interest is followed by deep sequencing; thereby, multiple mutations can be detected by CAPP-Seq including single nucleotide variants, rearrangements, and copy number alterations [93]. Implementing CAPP-Seq on blood samples of patients with early and advanced stage NSCLC, showed a high efficiency for detecting an allele frequency of *EGFR* mutations of down to 0.02% with >96% specificity [93,94]. Further improving the sensitivity of the CAPP-Seq, Newman et al. employed an integrated digital error suppression (iDES), a computational tool that can correct sequencing or PCR system error, resulting in a theoretical detection rate of 0.00025% mutant allele frequency [95]. iDES-enhanced CAPP-Seq has shown to be highly sensitive in the detection of *EGFR* mutations with an allele frequency as low as 0.004% with >99.99% specificity using cfDNA of NSCLC patients; furthermore, the required amount for the library preparation was only 32 ng [95], making it a very practical test for investigating ctDNA.

Although many advances have been made, NGS is still a relatively expensive and time-consuming technique. Furthermore, skilled bioinformaticians are required for data analysis and interpretation.

Bioinformatics are an essential part for the analysis of NGS to enable the detection of single nucleotide polymorphisms (SNPs), copy number aberrations (CNAs), insertions and deletions (indels), epigenetic changes, or to assembling new genomes [[96], [97], [98]]. The lack of standardization thus far, has led to the development of different algorithms performing essentially similar tasks in analyzing sequencing data, but using different mathematics. For instance, Burrows-Wheeler Alignment tool (BWA) [99], Bowtie [100], STAR [101], TopHap, and Novoalign are all short reads alignment tools [102]. Furthermore, variant calling can be performed using, e.g., GATK [103], SAM tools [104], Atlas2 [105], and FreeBayes [106]. In order to come to a possible consensus, the performance of these different tools must be regularly compared under different conditions. To assess the accuracy in variant calls, Bao et al. evaluated the four variant-calling algorithms, GATK-UnifiedGenotyper, SAMtools mpileup, Atlas2, and FreeBayes after alignment to the human genome using BWA, Bowtie2, and NovoalignV3. The authors used the NIST-GIAB gold standard dataset to demonstrate the sensitivities of these methods. Variant calls by FreeBayes from Novoalign V3 mapped sequences showed the highest sensitivity and precision rate for SNV calling of 95.97% and 99.70% and for indel calling 83.39% and 99.57%, respectively [102]. However, using simulated data, conflicting results were demonstrated by Kockan et al., indicating a low sensitivity and accuracy by using FreeBayes compared to SiNVICT, MuTect, and VarScan2 [107]. In the same study, the authors evaluated the sensitivity and accuracy of SiNVICT in the detection of SNVs and short indels of cfDNA. By analyzing two different datasets obtained from cfDNA sequenced material of castrate-resistant prostate cancer with Ion Torrent (AmpliSeq) technology and from metastatic castration-resistant prostate cancer patients sequenced with Illumina MiSeq, the SiNVICT demonstrated highly sensitive detection of variant calls at a low variant allele frequency of 0.5% [107]. These studies show that further investigation has to be performed in order to determine the most accurate methods for analyzing ctDNA.

### 4.2 Digital-PCR platforms

Digital PCR is a robust method to detect point mutations in ctDNA at low allele fractions. This technique includes droplet-based systems, microfluidic platforms for parallel PCR such as droplet digital PCR (ddPCR), and BEAMing (beads, emulsions, amplification, and magnetics).

Droplet-digital PCR (ddPCR) was developed to provide high-precision, absolute quantification of copy number variation of target DNA, such as quantification of somatic mutations [108]. The ddPCR approach is based on water-oil emulsion droplet technology by the distribution of DNA sample into thousands to millions of droplets. A single droplet contains a single mutated or non-mutated DNA strand that can be distinguished by flow cytometry using fluorescent TaqMan-based probes. ddPCR has been applied in several notable publications on the detection and quantification of mutations in ctDNA [78,109,110]. ddPCR demonstrated accurate detection of *PIK3CA* mutations in early stage breast cancer patients using ctDNA compared to tumor tissue with 93.3% sensitivity and 100% specificity [78]. Furthermore, Picodroplet digital PCR facilitates simultaneous screening for multiple mutations in ctDNA from the plasma with a detection rate of >1% [111]. The advantages of ddPCR are the high sensitivity in detecting mutations and as well as it being an inexpensive technology for absolute quantification. The disadvantages of ddPCR are that only known variants can be screened and the limited number of variants that can be investigated within a single reaction.

BEAMing is a digital PCR method that is based on beads, emulsion, amplification, and magnetics. This technology uses water droplets in an oil emulsion as reaction vessels containing a mixture of template, primers, PCR reagents, and magnetic beads. Fluorescently labeled dideoxynucleotide terminators are used to discriminate droplets containing sequences that diverge at positions of interest and analyzed by flow cytometry [112]. This technique can identify genetic variations present in the original DNA population and precisely quantify their number in comparison to the number of wild-type sequences [113]. BEAMing has shown a highly sensitive detection rate of 0.02% mutant allele frequency and a perfect specificity of 100%, with >90% concordance rate between tumor tissue and ctDNA from different patients with colorectal [35], breast [114], and lung [112,115,116] cancer. Although BEAMing is a highly sensitive and specific, its workflow is complicated and expensive to apply in routine clinical work.

### 4.3 Real-time PCR-based methods

Real-Time PCR represents a rapid and cheap method for amplification of nucleic acid. Its sensitivity to detect mutations in a background of wildtype DNA is 10–20% allele frequency, with almost no false positives [117,118]. To overcome the low sensitivity however, several PCR-based variations have been developed, such as Allele-Specific amplification (AS-PCR) [[119], [120], [121]], Allele-Specific Non-Extendable Primer Blocker PCR (AS-NEPB-PCR) [76], Peptide Nuclei Acid-Locked Nucleic Acid (PNA-LNA) PCR clamp [[122], [123], [124]], and co-amplification at lower denaturation temperature (COLD-PCR) [77]. Most of these assays are based on either using a blocking oligo at the 3′-end to block the amplification of the normal allele and allowing the amplification of the mutant allele or they make use of a modification step in the PCR protocol that enriches variant alleles from a mixture of wild-type and mutation-containing DNA. The AS-PCR is commonly used in clinical setting to detect single nucleotide variation (SNV) or small insertion/deletion in formalin-fixed, paraffin-embedded (FFPE) tumor tissues. However, as it exhibits 98% specificity and 92% sensitivity with a concordance of 96% of the mutant allele in ctDNA [119], it is not fully adequate for the detection of rare genetic events. The PNA-LNA PCR clamp method shows a high sensitivity with the detection of 0.1% mutant allele and a specificity of 79% [[122], [123], [124]]. COLD-PCR is a powerful method to detect single variants of approximately 0.1% and enables the enrichment of this amount of a mutant allele to improve the sensitivity of mutation detection by up to 100-fold [75,77]. Overall, PCR based assays are a promising tool for detecting mutations as a low-cost effective can be feasible in routine clinical practice.

### 4.4 Mass-spectrometry technology

The limited multiplexing ability of most PCR-based approaches represents a major limitation when dealing with clinical samples. Alternative technologies using mass-spectrometry have been developed to detect ctDNA mutations at low frequency, namely Surface-Enhanced Raman Spectroscopy (SERS) [125] and UltraSEEK [126,127].

The SERS-PCR detection method is based on using nanotags, which are nanoparticulate optical detection tags that function through surface-enhanced Raman Spectroscopy (SERS) for identification and tracking the binding target. Direct detection of multiple mutations at the same time using a Raman spectrometer is being enabled by laser excitation resulting in the emission of specific signals [128]. Multiplex PCR/SERS demonstrated high detection affinity of three hotspot mutations in melanoma showing a high sensitivity detection of <0.1% mutations with a low input amount of 5 ng DNA per reaction [125].

UltraSEEK is a high-throughput multiplex based method, using primers labeled with biotin that are specifically designed to anneal the mutant allele only [126]. The UltraSEEK assay panel covering the most frequent mutations in melanoma, showed a high sensitivity of detecting mutations at an allele frequency of <1% and a 100% specificity. Moreover, the minimum amount of cfDNA employed in the UltraSEEK analysis is between 9 pg/μl and 4.2 ng/μl [126]. Recently, the UltraSEEK's capacity has been further improved to a multiplexing of up to 40 targets per reaction, with ultrasensitive detection of somatic mutations in ctDNA [127]. Taken together, the advantages of UltraSEEK are the high multiplex capability, fast turnaround time of less than a day, and the low input of DNA required for a single analysis.

### 4.5 Detection of hypermethylation in ctDNA

Methylation of DNA involves the addition of a methyl group to CpG dinucleotides at regions of the genome with a high density of CpG dinucleotides or so-called CpG islands [7]. The most common method for methylation detection of ctDNA relies on methylation-specific PCR (MS-PCR), which is based on treating DNA with bisulfite to chemically modify non-methylated cytosines into uracil [71]. Subsequently, the methylation profile of the converted DNA can be investigated using a downstream application such as PCR, NGS [129], or MCTA-Seq [130]. Methylation-specific PCR (MS-PCR) has shown to be highly sensitive in the detection of *ESR1* hypermethylation with a detection rate of 0.1% and a specificity of 100% [71]. Higher sensitivities may be reached by MCTA-Seq, which is able to detect thousands of hypermethylated CpG islands in parallel with a sensitivity of detecting methylated CpG alleles down to frequencies of <0.25%, but with a specificity of 89%. Nevertheless, the input amount of ctDNA of 7.5 pg is a clear advantage [130]. The costs, processing time, and the requirement of prior knowledge of the region of interest are disadvantages of MCTA-Seq. A genome-wide bisulfite sequencing for the identification of different methylated regions using >500 ng urinary cfDNA starting material, could show that the global methylation density in cancer is ranging from 61.1% to 73.5% [129]. However, the relatively large amount of 500 ng cfDNA that is required for the bisulfite conversion process increases the complexity of the methylation detection using ctDNA from plasma [129].

## 5 Outlook

As this review indicates, numerous studies have now shown the feasibility of using ctDNA in tracking and monitoring tumor dynamics, drug response, and therapy resistance. Although several technologies have shown an extremely high sensitivity with detection rates going down to single mutated DNA molecules, the use of ctDNA as a marker for liquid biopsy still lacks standardization in many aspects. The only tests thus far approved by the FDA in the USA and China include the DNA methylation-based test of *SEPT9* for the detection of colorectal cancer [131,132] and the qPCR-based test for mutated *EGFR* in NSCLC [133]. Further improvement in the standardization of liquid biopsy may include how the samples are obtained and how the analysis is performed.

Ideally, ctDNA should be investigated in combination with CTCs and/or exosomal miRNA, in order to extract as much biological information from the tumor as possible from a single blood sample. However, the type of collection tube and storage conditions may both have an effect on DNA stability as well as the stability of cells and thereby the amount of background and the quality of the material. Although fixatives may stabilize a tube's content required for transport of the material, not every fixative suitable for subsequent cellular or DNA analysis can be used in combination with RNA analysis. Also, too harsh fixation conditions can result in DNA interstrand crosslinking and thereby lowering the specificity of downstream analyses. It needs to be seen whether there will be one standard tube from which all analyses can be performed, although more likely will be that each biomarker will require its own dedicated collection tube.

An important aspect of mutation diagnostics, not limited to the analysis of ctDNA only, is the sheer amount of data that can be produced by current technologies such as NGS, which can be overwhelming from a clinical point of view. However, bioinformatic-based techniques are usually able to filter out the clinically most important information. Nevertheless, also standardization in regards of bioinformatic analysis needs to be achieved in order for such diagnostics to be reliably be applied in the clinic.

As discussed in this review, one of the hurdles of using ctDNA as liquid biopsy substrate is the usually low yield of material extracted from plasma. In order to obtain enough starting material for further downstream analyses such as deep sequencing, whole genome amplification (WGA) might be employed. However, further research has to be performed to study whether the currently available WGA methods are suitable for highly fragmented DNA, as well as whether the amplification is perfectly linear so that low frequency alleles are not lost.

Understanding the biological mechanisms of how ctDNA is released into the bloodstream may further improve the isolation of the tumor DNA as well as prognosis and prediction value. For instance, the specific enrichment of tumor-associated exosomes may provide undiluted information about potential metastatic sites and the resistance mechanisms of the still viable tumor cells under therapy. Equally important is to investigate the elimination rate of cfDNA from the bloodstream. Several mechanisms and organs appear to be responsible for cfDNA clearance from the bloodstream such as the kidney, liver, and spleen as well as nuclease degradation, and phagocytes [[134], [135], [136]]. Nevertheless, the kinetic dynamics of cfDNA still needs to be further investigated, as well as the best source of ctDNA, e.g., serum, plasma, urine, or other body liquids should be standardized.

ctDNA can play a vital complementary role along with other tumor-derived substrates as predictive biomarker. These other substrates include circulating tumor cells (CTCs) that provide essential information on tumor characteristics and metastatic development through investigation of DNA, RNA, or proteins, whereas cell-free nucleotides and exosomes can be an additional sources of information on tumorigenesis, possible therapeutic targets, and drug resistance mechanisms. Finally, platelets can carry information that may help to determine the tumor's origin. Overall, these tumors-substrates termed as liquid biopsy that can provide a more comprehensive picture together of the total clonal composition of tumor and therapy sensitivity and thereby, improve on clinical management and patient survival.

## 6 Conclusion

Liquid biopsy can provide valuable information about the biology and clinical characteristics of a tumor through different biomarkers released into the blood circulation. ctDNA can be employed to analyze the entire tumor genome and track drug response and/or therapy resistance. This can be achieved by either quantitative measurement of ctDNA in a blood sample or by the detection of mutations. A remarkable advancement in technologies for ctDNA detection and analysis has been observed in the last few years such as the significant progress made in NGS-based approaches in overcoming many of the challenges to reduce the error rate and improve sensitivity in ctDNA detection. Nevertheless, NGS-based approaches are still relatively expensive and consume much time. On the other hand, mass-spectrometry approaches provide a promising tool for ctDNA screening in terms of the cost, time, and low amounts of required input material, as well as their high sensitivity and specificity. Additionally, analysis by Real-Time PCR-based techniques is cost-effective, fast, and can be feasible in routine clinical practice for a limited number of biomarkers. Further development in the standardization of these techniques will make ctDNA a valuable substrate in the field of cancer diagnostics.

## Acknowledgements

Financial support by the Erich & Gertrud Roggenbuck Foundation.
